# The modification and characterization of thermal-treated sericite by fluorosilicate

**DOI:** 10.1038/s41598-018-32496-x

**Published:** 2018-09-24

**Authors:** Yu Liang, Wei Jiang, Hao Ding, Yongkui Wang

**Affiliations:** 1 Beijing Key Laboratory of Materials Utilization of Nonmetallic Minerals and Solid Wastes, National Laboratory of Mineral Materials, School of Materials Science and Technology, China University of Geosciences (Beijing), Beijing, 100083 China; 2 School of Materials Science and Technology, Shenyang University of Chemical Technology, Shenyang, 110142 Liaoning Province China

## Abstract

In this article, the thermal-treated sericite was modified by both fluorosilicate and the combination of fluorosilicate and nitric acid in order to reduce its layer charge and gain cation exchange capabilities for the preparation of sericite/polymer nanocomposites. After several orthogonal experiments and single factor experiments, the optimal experimental conditions were set up and we found that the combination of nitric acid and fluorosilicate is much more effective than fluorosilicate alone. Chemical composition analysis showed Al^3+^ was dissolved out from sericite and the dissolving amount is 65 mg/g under optimal experimental conditions. Combining the NMR test, it is considered that the Si/Al ratio in the tetrasheet of the modified product increased from 3.48 to 10. The layer charge reduced and the CEC value increased after fluorosilicate modification, which means the modified sericite is a promising matrix for clay-polymer nanocomposites.

## Introduction

Sericite is a kind of 2:1 phyllosilicate mineral similar to muscovite in structure and chemical composition. It has a net negative surface charge induced by the substitution of Al^3+^ for Si^4+^ in tetrahedral sheets, which was balanced by K^+^, fitting in quite compactly. Therefore, it has almost no ion exchange capacity and is not expandable.

Sericite has several unique properties compared with other layered silicates. It has higher resiliency than other fillers and can shield and absorb ultraviolet radiation. It also has a high aspect ratio, with its value greater than 1000^1–3^. Therefore, it is a good choice to use sericite to prepare polymer/layered silicate (PLS) nanocomposites with improved light stability and excellent barrier properties. If sericite can be fully exfoliated, the aspect ratio of its individual layer will exceed the layer of montmorillonite in the matrix to a large extent^4^.

Genarally, several kinds of clays were used to prepare polymer/layered silicate (PLS) nanocomposites such as montmorillonite^5–8^, kaolinite^9–12^ and vermiculite^13–16^. The polymer matrix can be epoxies^17–20^, elastomers^21,22^, polyactide^23^ and polypropylene^24,25^. Since sericite has no ion exchange capacity and no expandability in water^26^, it can hardly be intercalated and was rarely used in nanocomposites. Therefore, it is necessary to explore the preparation of an expandable sericite with relatively high cation exchange capacity (CEC) in order to prepare sericite/polymer nanocomposites.

The key to obtaining cation exchange abilities of sericite is to reduce the charge quantity of its structure layer, which can be realized by reducing Al/Si ratio. Therefore, the purpose of modification is to permanently reduce the layer charge of sericite and obtain a number of exchangeable cations. Fluorosilicate is a kind of compound with abnormal solubility and is usually used as pesticide and disinfectant^27^. In the lab, it was used to prepare high silicon Y-type zeolite by dissolving aluminum out from lattice and inserting silicon inside the corresponding holes^28^. Compared with other methods, this method has the advantages of maintaining its original structural stability and producing less frame hydroxide holes^29^. If fluorosilicate is used to modify phyllosilicate, it is expected that it will improve the silicon/aluminum ratio in a similar way, reducing the layer charge and improving the cation exchange capacity accordingly. Other physical and chemical modifications, such as acid activation^30–32^, mechanical grinding^33,34^ and thermal modification^35^ have long been used to activate clay and clay minerals. F.J. del Rey-Perez-Caballero^13^ used acid treatment followed by calcination to treat vermiculate and reduced global negative charge of vermiculate. The negative charges of phlogopite were also reduced in a similar way^36,37^.

In this article, the thermal-treated sericite was used as raw materials and it was modified both by fluorosilicate and by the combination of fluorosilicate and nitric acid. The modification effect was judged by the amount of dissolved aluminum, structure characterization and other characterization methods. The experimental conditions were optimized by several orthogonal experiments and single factor experiments. The fluorosilicate modification reduced its layer charge and improved the CEC value of sericite, which will definitely, create conditions for the preparation of polymer-sericite nanocomposites with improved properties than others.

## Materials and Methods

### Materials

The raw sericite (S_0_) was obtained from Anhui province, China with its mean size about 10 μm. The chemical composition of S_0_ is listed in Table 1. From its chemical composition, it can be seen that the content of K_2_O is close to the content of K_2_O in pure sericite, which means the raw sericite has a rather high purity.

**Table 1:** Chemical composition of raw sericite.

Composition	SiO_2_	Al_2_O_3_	K_2_O	Na_2_O	Fe_2_O_3_	FeO	TiO_2_	MgO	CaO	MnO	H_2_O	L.O.I
ωB(%)	47.30	30.02	10.37	0.45	2.02	0.41	0.64	1.53	0.22	0.040	0.26	4.19

The raw sericite was first heated to 800 °C in a furnace for 1 h. The thermal-treated sericite (S_1_) was allowed to cool down to room temperature and was used as a raw material in this experiment.

### Preparation

Sodium fluorosilicate was dissolved in the suspension of thermal-treated sericite and the whole system was heated at a certain temperature for some time. After that, the modified sericite was filtrated and dried. In this study, experiment term was based on an orthogonal term array experimental design (OA (9, 3^4^)) where the following four variables were analyzed: the concentration of fluorosilicate (factor A), reaction temperature (factor B), reaction time (factor C) and solid-to-liquid ratio (factor D). In the case of the combination of nitric acid and sodium fluorosilicate co-modification, both nitric acid and sodium fluorosilicate were added into the suspension of thermal-treated sericite and the whole system was heated at a certain temperature for some time. After that, the modified sericite was filtrated and dried. In this study, the solid-to-liquid ratio was fixed as 3:200 and the experiment term was based on an orthogonal term array experimental design (OA (9, 3^4^)) where the following four variables were analyzed: the concentration of fluorosilicate (factor A), reaction temperature (factor B), reaction time (factor C) and the volume of nitric acid (factor D).

### Characterization

The X-ray diffraction patterns were obtained on a Rigaku Rotaflex X-ray powder diffractometer (Rigaku, Tokyo, Japan), employing Cu Kα radiation, 40 kV, 100 mA. The X-ray diffraction (XRD) patterns in the 2θ range from 3°–70° were collected at 4°/min. ^27^Al NMR spectrum (130.327 Hz) was recorded on a Bruker Avance III spectrometer (Bruker, Karlsruher, Germany). The dwell time is 0.01 s and the rotational speed is 6000 rpm. Zeta potential tests were done on Zeta PALS (Brookhaven Instruments, Co., Holtsville, USA) with pH ranging from 2 to 12.

## Results and Discussion

### The modification of thermal-treated sercite by fluorosilicate only

As to the modification of thermal-treated sericite by fluorosilicate, the main four factors, the concentration of fluorosilicate (factor A), reaction temperature (factor B), reaction time (factor C) and solid-to-liquid ratio (factor D) were researched and each control parameter has three experimental levels (Table 2)^38,39^. The modification effect was evaluated by the dissolving-out amount of Al^3+^. The dissolution of Al^3+^ and the reduction of negative layer charge allow sericite to show ion exchange capacity. Usually, the larger the dissolving amount of Al^3+^, the better the modification effect.

**Table 2:** Design and results of the orthogonal experiment of fluorosilicate modification of sericite^a^.

Trail No.	Factors				Results: Dissolving-Out Amount of Al^3+^ (mg/g)
	Fluorosilicate ConcentrationA (mol/L)	Reaction Temperature B (°C)	Reaction Time C (h)	Solid-to-liquid ratio D (g:ml)	
A101	0.025	40	1	4:200	2.5545
A102	0.025	65	3	7:200	1.2969
A103	0.025	90	5	10:200	0.7074
A104	0.05	40	3	10:200	0.8646
A105	0.05	65	5	4:200	2.5545
A106	0.05	90	1	7:200	1.0106
A107	0.075	40	5	7:200	1.460
A108	0.075	65	1	10:200	1.0218
A109	0.075	90	3	4:200	2.1615
K_1*j*_	4.5588	4.8791	4.5869	7.2705	—
K_2*j*_	4.4292	4.8732	4.323	3.7675	—
K_3*j*_	4.6433	3.8795	4.7219	2.5938	—
ω_1*j*_	0.005	0.1118	0.0144	0.9089	—
ω_2*j*_	−0.0382	0.1098	−0.0736	−0.2588	—
ω_3*j*_	0.0331	−0.2214	0.0594	−0.65	—
R_*j*_	0.0713	0.3332	0.133	1.5589	—

^a^K_ij_ is defined as the sum of the evaluation indexes of all levels (i, i = 1, 2, 3) in each factor (j, j = A, B, C, D) and ω_ij_ (mean value of K_ij_) is used to determine the optimal level and the optimal combination of factors. The optimal level for each factor could be obtained when ω_ij_ is the largest; R_j_ is defined as the range between the maximum and minimum value of ω_ij_ and is used for evaluating the importance of the factors.

According to Table 2, the factors’ levels of significance are as follows: solid-to-liquid ratio > reaction temperature > reaction time > fluorosilicate concentration. When the concentration of sodium fluorosilicate increased from 0.025 mol/L to 0.05 mol/L until 0.075 mol/L, the relevant influential effect decreased first, then increased, while the total influential effect was quite trivial. Considering the slight solubility of sodium fluorosilicate, 0.025 mol/L was chosen as the optimal fluorosilicate concentration.

Similarly, when the reaction time increased from 1 h to 3 h until 5 h, the relevant influential effect decreased first, then increased, while the total influential effect was quite trivial. Therefore, 1 h was chosen as the optimal reaction time. The optimal solid-to-liquid ratio is 4:200 (g:ml) and the optimal reaction temperature is 40 °C. Considering all the factors, trail number A101 is the combination of optimal reaction conditions and the dissolving-out amount of Al^3+^ reached 2.5545 mg/g.

**Figure 1:**
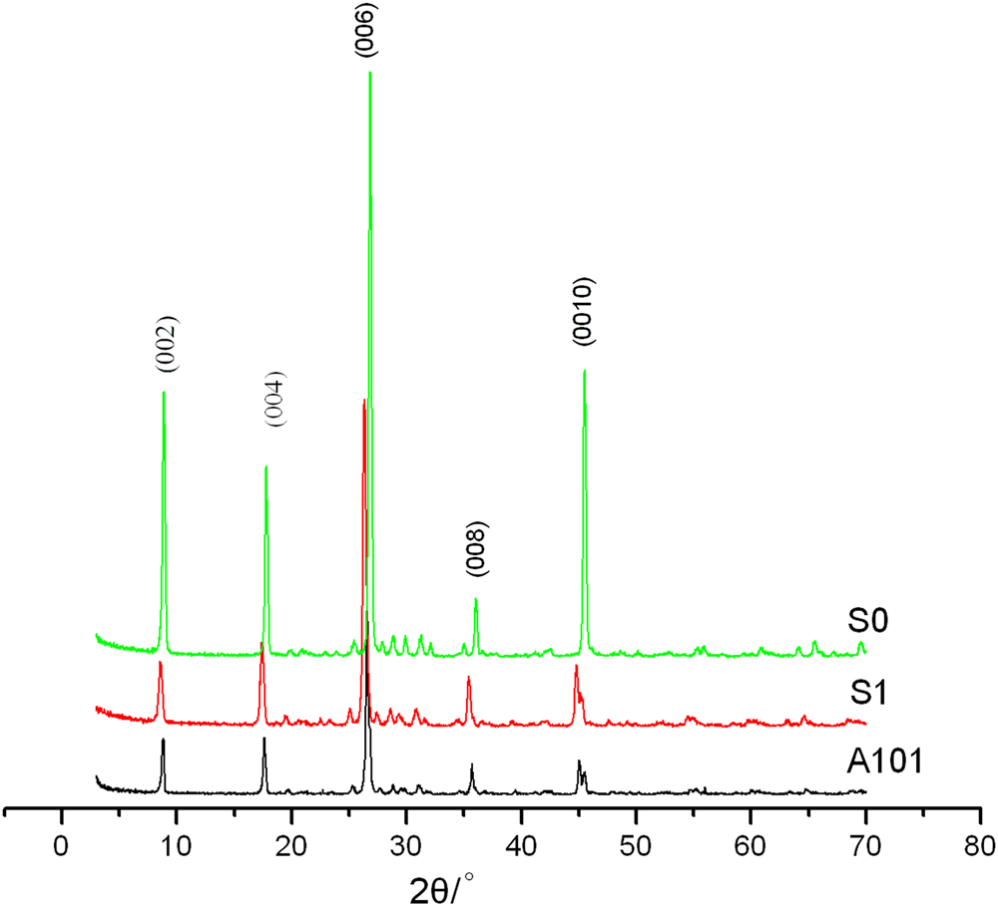
XRD patterns of raw sericite (S_0_), thermal-treated sericite (S_1_) and fluorosilicated sericite (the optimal combination of reaction conditions, A101).

Figure 1 shows the XRD patterns of the raw sericite (S_0_), thermal-treated sericite (S_1_) and fluorosilicated sericite (the optimal combination of reaction conditions) and Table 3 shows the relevant data.

**Table 3:** XRD data of the related products.

Trial No.	2θ(°)	d_002_(nm)	I_max_	FWHM
S_0_	8.905	0.992	10643	0.34
S_1_	8.630	1.024	2406	0.48
A101	8.846	0.999	2248	0.31

Compared with raw sericite (S_0_), the thermal-treated sericite (S_1_) still kept complete structure of sericite, while the intensities of major reflections decreased and FWHM increased accordingly, which means that the raw sericite was activated after thermal modification. The thermal-treated sericite is more suitable to be used for the subsequent modification. After fluorosilicate modification, the intensity of corresponding peaks reduced further, while complete crystal form still kept. From Table 3, it can be seen that the FWHM of fluorosilicated sericite decreased, which indicated that the dissolving-out of Al^3+^ decreased the crystallization degree of sericite while increasing the symmetry degree.

A second orthogonal experiment was done based on the results of the first orthogonal experiment in order to have more detailed information about the relevant reaction factors. Four factors, the concentration of fluorosilicate (factor A), reaction temperature (factor B), reaction time (factor C) and solid-to-liquid ratio (factor D) were researched and each control parameter has three experimental levels (Table 4).

**Table 4:** Design and results of the second orthogonal experiment of fluorosilicate modification of sericite^.^

Trial No.	Factors				Results: Dissolving-Out Amount of Al^3+^ (mg/g)
	Fluorosilicate ConcentrationA (mol/L)	Reaction Temperature B (°C)	Reaction Time C (h)	Solid-to-liquid ratio D (g:ml)	
A201	0.015	30	0.5	3:200	1.648
A202	0.015	40	1	4:200	1.173
A203	0.015	50	1.5	5:200	0.8664
A204	0.025	30	1	5:200	1.4436
A205	0.025	40	1.5	3:200	2.045
A206	0.025	50	0.5	4:200	1.98375
A207	0.035	30	1.5	4:200	1.62375
A208	0.035	40	0.5	5:200	1.227
A209	0.035	50	1	3:200	2.526
**K**_**1*j***_	3.6874	4.7154	4.8588	6.219	—
**K**_**2*j***_	5.4724	4.445	5.1426	4.7805	—
**K**_**3*j***_	5.3768	5.3762	4.5352	3.537	—
**ω**_**1*j***_	−0.3861	−0.0434	0.0044	0.4578	—
**ω**_**2*j***_	0.2089	−0.1335	0.099	−0.0217	—
**ω**_**3*j***_	0.1771	0.1769	−0.1035	−0.4362	—
**R**_***j***_	0.595	0.3104	0.2025	0.894	—

According to Table 4, the factors’ levels of significance are as follows: solid-to-liquid ratio > fluorosilicate concentration > reaction temperature > reaction time. Solid-to-liquid ratio is still the most significant factor, which corresponds to the results in the first orthogonal experiment, while other factors’ influence order has changed. According to the results, the optimal combination of reaction conditions is A2B3C2D1, that is fluorosilicate concentration 0.025 mol/L, reaction temperature 50 °C, reaction time 1 h and solid-to-liquid ratio 3 g: 200 ml. Since no experiment was done using an optimal reaction condition, an extra experiment was conducted and it was found that the dissolving-out amount of Al^3+^ reached 2.181 mg/g under such optimal reaction condition.

**Figure 2:**
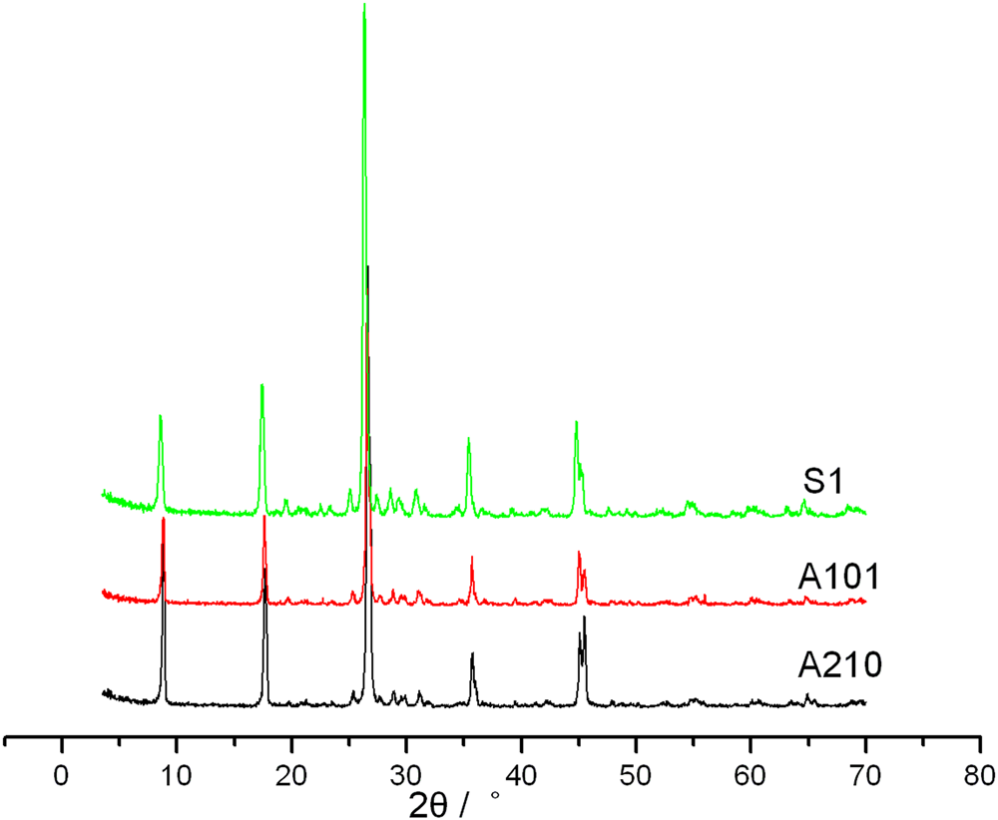
XRD patterns of thermal-treated sericite (S_1_) and fluorosilicated sericites (A101 is the optimal product in the first orthogonal experiment and A210 is the optimal product in the second orthogonal experiment).

Figure 2 shows the XRD patterns of thermal-treated sericite (S_1_) and fluorosilicated sericites (A101 is the optimal product in the first orthogonal experiment and A210 is the optimal product in the second orthogonal experiment) and Table 5 shows the relevant data.

**Table 5:** XRD data of the related products.

Trial No.	2θ(°)	d_002_(nm)	I_max_	FWHM
S_0_	8.905	0.992	10643	0.34
S_1_	8630	1.024	2406	0.48
A101	8.846	0.999	2248	0.31
A210	8.879	0.951	4605	0.21

From the XRD patterns it can be seen that both A210 and A101 still kept the crystalline form of sericite, which means that sodium fluorosilicate didn’t have substantial destruction on the structure of sericite while dissolving Al^3+^ out. This provides guarantee for further modification and application of sericite with its own mineral properties remained. Compared with S_0_, both the intensities of (002) crystal plane of A210 and A101 decreased by a large margin. However, when compared with S_1_, it can be seen that the intensities of A101 decreased slightly while the intensities of A210 increased instead, which means the usage of sodium fluorosilicate restored the integrity of sericite as well as dissolving Al^3+^ out in the second orthogonal experiment. Meanwhile, the FWHM of A210 decreased compared with A101. These results show that A210 is better than A101.

### The modification of thermal-treated sericite by the combination of fluorosilicate and nitric acid

From the above experiment results, it can be seen that the modification of fluorosilicate can dissolve Al^3+^ out from sericite, however, the dissolving amount of Al^3+^ is quite limited (only 2.181 mg/g under an optimal reaction condition), which of course, can hardly reduce the layer charge of sericite to the ideal state. Therefore, the combination of fluorosilicate and nitric acid was used to modify thermal-treated sericite. First, an orthogonal experiment was done with four main factors at three levels (Table 6). According to the above experiment, it can be seen that the smaller the solid-to-liquid ratio, the better the result. Therefore, the solid-to-liquid ratio was set as 3:200 (g:ml) in this experiment. Still, the modification effect was evaluated by the dissolving-out amount of Al^3+^.

**Table 6:** Design and results of the orthogonal experiment of the combination modification of fluorosilicate and nitric acid.

Trail No.	Factors				Results: Dissolving-Out Amount of Al^3+^ (mg/g)
	Fluorosilicate Concentration A (mol/L)	Reaction Temperature B (°C)	Reaction Time C (h)	Nitric acid volume D (ml)	
H101	0.025	40	1	10	6.27
H102	0.025	65	3	30	25.989
H103	0.025	90	5	50	120.403
H104	0.05	40	3	50	13.721
H105	0.05	65	5	10	36.893
H106	0.05	90	1	30	57.248
H107	0.075	40	5	30	21.809
H108	0.075	65	1	50	27.261
H109	0.075	90	3	10	99.957
K_1*j*_	152.662	41.800	90.779	143.120	—
K_2*j*_	107.862	90.143	139.667	105.046	—
K_3*j*_	149.027	277.608	179.105	161.385	—
ω_1*j*_	5.381	−31.573	−15.246	2.201	—
ω_2*j*_	−9.552	−15.458	1.050	−10.491	—
ω_3*j*_	4.170	47.03	14.196	8.289	—
R_*j*_	14.933	78.603	29.442	18.780	—

According to Table 6, the factors’ levels of significance are as follows: reaction temperature > reaction time > nitric acid volume > fluorosilicate concentration. The most significant factor is reaction temperature. The optimal combination of reaction conditions is A1B3C3D3, that is fluorosilicate concentration 0.025 mol/L, reaction temperature 90 °C, reaction time 5 h and nitric acid volume 50 ml. The joint action of both fluorosilicate and nitric acid can dissolve large amount of Al^3+^ from sericite (the dissolving-out amount of Al^3+^ is 120.403 mg/g), while these two factors restrained each other, which can be seen from the effect of the volume of nitric acid. Nevertheless, the combination modification effect of fluorosilicate and nitric acid is much more remarkable than fluorosilicate itself (the dissolving-out amount of Al^3+^ by fluorosilicate alone was 2.5545 mg/g).

**Figure 3:**
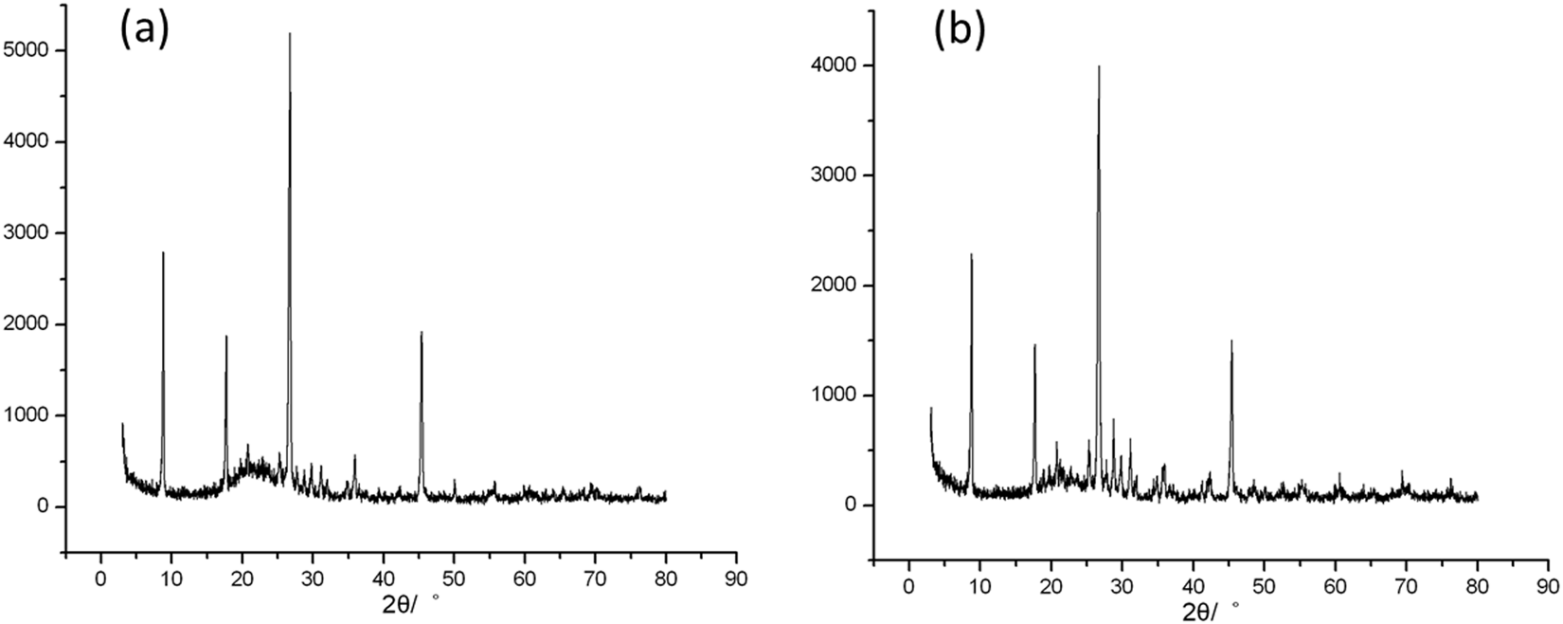
XRD patterns of **(a**) H103 (low concentration of fluorosilicate and high concentration of nitric acid product) and (**b**) H109 (high concentration of fluorosilicate and low concentration of nitric acid product).

XRD patterns of the above 9 products shows that they all kept crystal state, while the intensities and the shapes of the peaks changed at different levels. Among them, H103 (low concentration of fluorosilicate and high concentration of nitric acid) and H109 (high concentration of fluorosilicate and low concentration of nitric acid) all have high dissolving amount of Al^3+^, while their XRD patterns have wide background, which means that amorphous substances have emerged after modification (Fig. 3).

Table 7 shows the crystallinity degree and the relative crystallinity degree of the products. Both the crystallinity degree of H103 and H109 declined obviously compared with A210, with the relative crystallinity degree at about 60%, which means the modification by the combination of fluorosilicate and nitric acid in high concentration can dissolve large amount of Al^3+^ out as well as destroy the integrity of crystal form seriously. Although the CEC value of H109 after sodium chloride modification is 9.40 mmol/100 g, higher than raw sericite (4.94 mmol/100 g), still it is not that much, which confirms the modification effect is not that ideal. Therefore, neither the concentration of fluorosilicate nor the concentration of nitric acid should be too high.

**Table 7:** The crystallinity degree and the relative crystallinity degree of the related products.

Trail No.	S_0_	S_1_	A210	H103	H109
crystallinity degree	0.699	0.576	0.624	0.396	0.461
relative crystallinity degree	1.000	0.824	0.893	0.567	0.660

**Figure 4:**
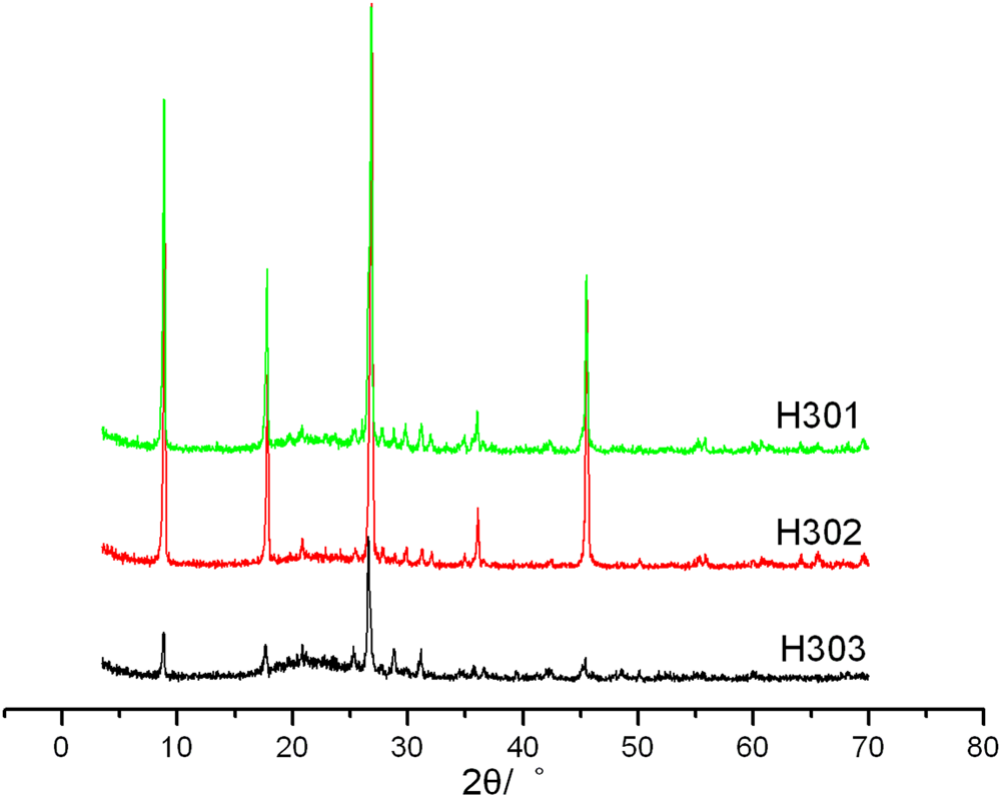
XRD patterns of different samples with different volume of nitric acid (H301: 10 ml nitric acid; H302: 20 ml nitric acid; H303: 30 ml nitric acid).

Considering the importance of fluorosilicate and nitric acid in this experiment, single factor experiments were conducted with these two factors. Table 8 showed the effect of nitric acid volume on the product. Other experiment conditions were set as: the concentration of fluorosilicate 0.025 mol/L, reaction temperature 90 °C, reaction time 5 h and solid-to-liquid ratio 3 g/200 ml. When the volume of nitric acid was 30 ml, the dissolving amount of Al^3+^ reached 133.002 mg/g, with a large increasement compared with the other two. XRD patterns show that all the three samples kept the crystal form of sericite, while the intensities and the shapes of peaks changed (Fig. 4). This is especially true for H303, with its peak intensities declined obviously and background grew wider than the other two, which means that more amorphous substances have emerged in H303 after modification.

**Table 8:** The effect of nitric acid volume to the dissolving-out amount of Al^3+^

Trial No.	H301	H302	H303
Nitric acid volume (ml)	10	20	30
Dissolving-out amount of Al^3+^(mg/g)	85.958	80.630	133.002

From the data in Table 9, it can be seen that the relative crystallinity of H303 is much lower than H301 and H302 (only 0.492). Among the three products, H301 has the largest intensity of (002) peak together with smaller FWHM, which means the crystal structure of H301 was kept much better than the other two after the modification. Therefore, the optimal usage of nitric acid is 10 ml.

**Table 9:** XRD data of the related products (single factor experiment of nitric acid volume).

Trail No.	2θ(°)	d_002_(nm)	I_max_	FWHM	Crystallinity	Relative crystallinity
S_0_	8.905	0.992	10643	0.34	0.699	1.000
S_1_	8.630	1.024	2406	0.48	0.576	0.824
H301	8.883	0.995	6056	0.20	0.511	0.731
H302	8.936	0.989	5513	0.20	0.590	0.844
H303	8.858	0.997	794	0.25	0.344	0.492

Table 10 shows the effect of fluorosilicate concentration to the product. Other experiment conditions were set as: the volume of nitric acid 10 ml, reaction temperature 90 °C, reaction time 5 h and solid-to-liquid ratio 3 g/200 ml. It can be seen that the dissolving-out amount of Al^3+^ increased with the concentration of fluorosilicate.

**Table 10:** The effect of fluorosilicate concentration to the dissolving-out amount of Al^3+^.

Trail No.	H401	H402	H403	H301
Fluorosilicate concentration (mol/L)	0.01	0.015	0.02	0.025
Dissolving-out amount of Al^3+^(mg/g)	52.570	65.000	67.670	85.958

XRD tests show that all the three products kept crystal form with relatively high crystal state (Table 11), much better than H301. Among them, H402 has the largest (002) peak intensities together with the lowest FWHM, which indicates its best crystal integrity among these products. Therefore, the optimal experimental conditions are: reaction temperature 90 °C, reaction time 5 h, solid-to-liquid ratio 3 g/200 ml, the volume of nitric acid 10 ml and the concentration of fluorosilicate 0.015 mol/L. The optimal product has 65 mg/g Al^3+^dissolved out after the modification by the combination of nitric acid and fluorosilicate.

**Table 11:** XRD data of the related products (single factor experiment of fluorosilicate concentration).

Trail No.	2θ(°)	d_002_(nm)	I_max_	FWHM	Crystallinity	Relative crystallinity
S_0_	8.905	0.992	10643	0.34	0.699	1.000
S_1_	8.630	1.024	2406	0.48	0.576	0.824
H401	9.001	0.987	4232	0.28	0.639	0.914
H402	8.889	0.994	5348	0.22	0.635	0.908
H403	8.854	0.998	5149	0.25	0.614	0.878
H301	8.883	0.995	6056	0.20	0.511	0.731

### The properties of the modified sericite

The chemical composition analysis was done for both raw sericite (S_0_) and fluorosilicated sericite by the combination of nitric acid and sodium fluorosilicate (S_2_) (Table 12). Compared with S_0_, the SiO_2_ contents and Na_2_O contents increased while the content of Al_2_O_3_ decreased in S_2_. The modification by the combination of nitric acid and fluorosilicate can dissolve Al^3+^ out from sericite, which of course, will improve Si/Al ratio to a large extent. The increase of Na_2_O content was induced by the Na^+^ in the sodium fluorosilicate by ion exchanges. Other oxide contents, such as Fe_2_O_3_, FeO and K_2_O, decreased.

**Table 12:** The chemical analysis of sericite before and after modification.

Trail No.	Main Composition (*ω*_B_/%)									
	SiO_2_	Al_2_O_3_	Fe_2_O_3_	FeO	TiO_2_	K_2_O	Na_2_O	MgO	CaO	MnO
S_0_	47.30	30.02	2.02	0.41	0.64	10.37	0.45	1.53	0.22	0.040
S_2_	71.92	17.14	1.14	0	0.29	4.04	1.39	1.30	0.03	0.013

The filtrate during the modification was also detected in order to know the dissolution of different components from sericite (Table 13). Large amount of Al^3+^ and K^+^ have been dissolved together with Fe^3+^ and Mg^2+^ during the modification. Since sodium fluorosilicate also contains Na^+^, therefore, the detection of Na^+^ couldn’t reflect the dissolution of Na^+^ from sericite. This is in accordance with the chemical analysis of sericite after modification.

**Table 13:** Ion contents from the filtrate during modification.

Cation	Si^4+^	Na^+^	Al^3+^	Fe^3+^	K^+^	Mg^2+^
Dissolving-out amount (mg/g)	0.89	40.25	65.00	4.61	38.12	5.09

**Figure 5:**
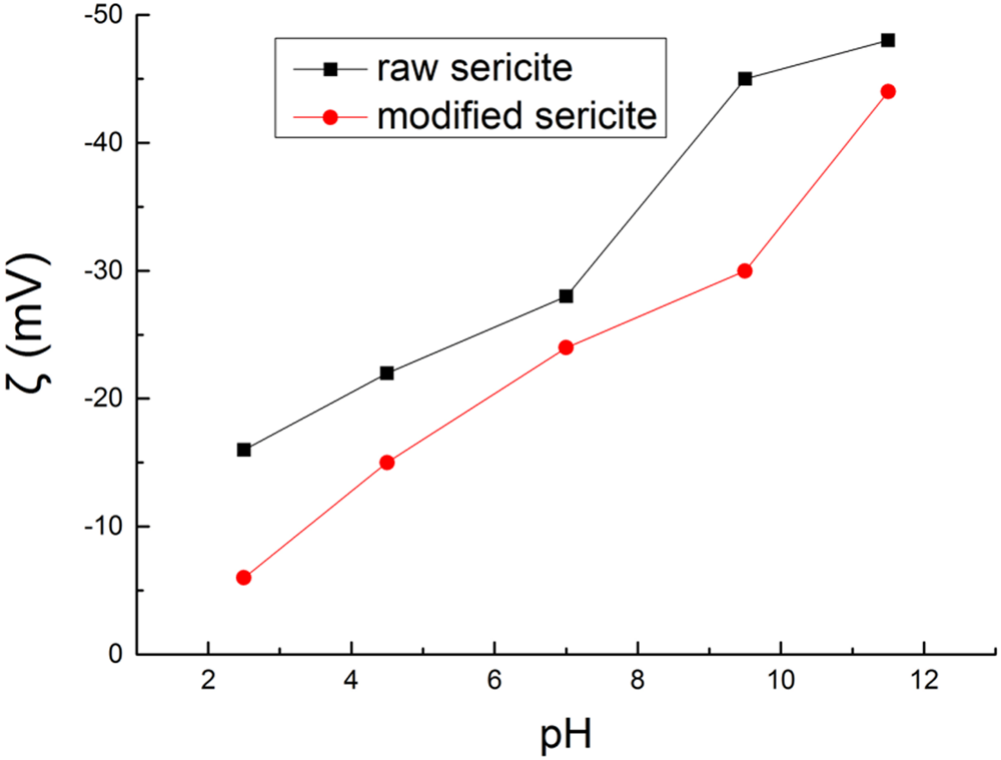
Zeta potential of raw sericite and fluorosilicate modified sericite.

Zeta potential tests (Fig. 5) were conducted for sericite before and after modification. Both zata potential of raw sericite and modified sericite decreased with the increasing pH value of the solution. The absolute zeta potential value of the modified sericite was always lower than raw sericite, which means that the modification reduces the layer charge of sericite. This is also in accordance with data in Table 12 and Table 13, which shows that the modification dissolves Al^3+^ out from sericite. The substitution of Si^4+^ by Al^3+^ in the tetrahedral sheet leads to the negative interlayer charge and makes sericite have no ion exchange capacity. After fluorosilicate modification, the layer charge of sericite decreased, which indicated that the modified sericite may gain cation exchange capacity, which of course, will help it to be used in the sericite/polymer nanocomposites.

CEC value was tested in order to evaluate the cation exchange capacities of sericite before and after modification (Table 14). The CEC value of raw sericite is 4.94 mmol/100 g, which is caused by the unsaturated bonds on the surfaces and ends during mineral processing and chemical analysis (Table 12) shows that a certain amount of Na^+^ exists in the interlayer places. After fluorosilicate modification, the CEC value of S_2_ increased to 14.84 mmol/100 g, which means the fluorosilicate modification improved the cation exchange capacities and the structure of sericite. This result is also in accordance with the zeta potential tests before.

**Table 14:** CEC value of raw sericite and fluorosilicated sericite.

	Raw sericite (S_0_)	Sericite modified by the combination of fluorosilicate and nitric acid (S_2_)
CEC(mmol/100 g)	4.94	14.84

### The fluorosilicate modification mechanism of sericite

**Figure 6:**
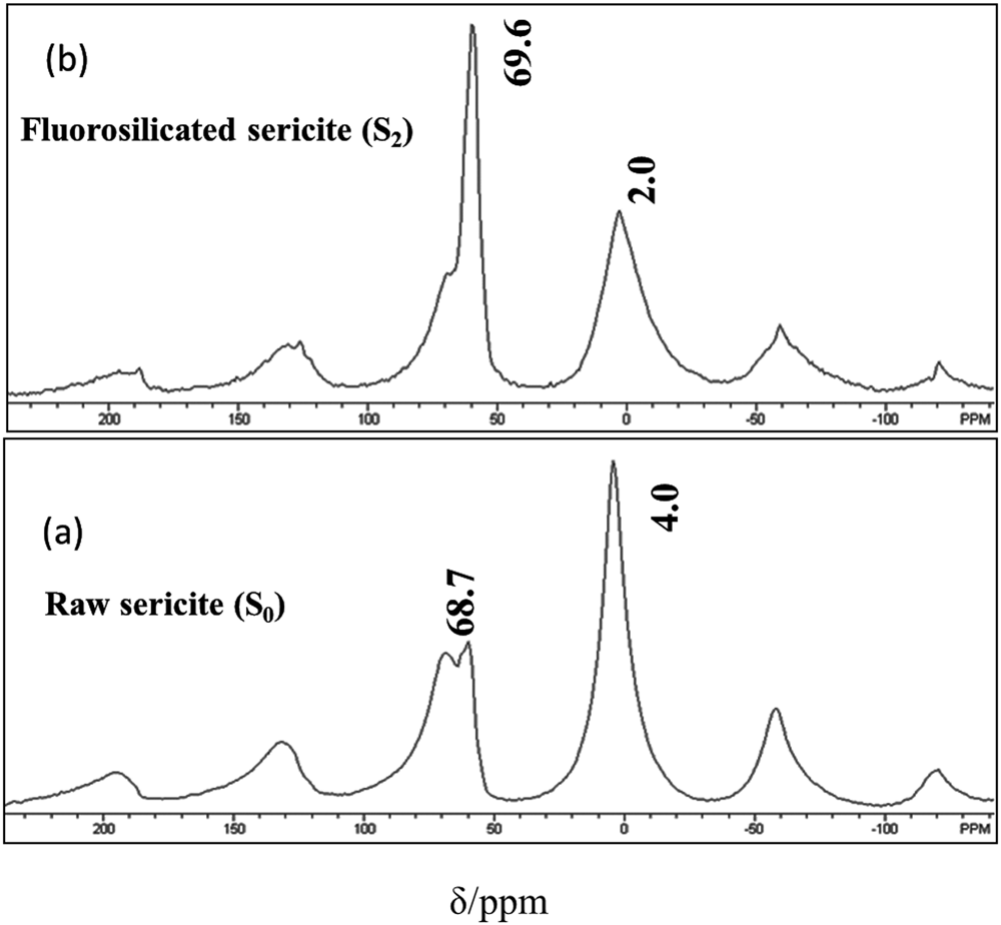
^27^Al NMR spectrums of S_0_ and S_2._

NMR analysis was done after fluorosilicate modification. The range of chemical shift (δ) of Al is 450 ppm. Generally, δ of octahedral Al (Al_o_) species and tetrahedral Al (Al_t_) species is −10 to 10 ppm and 50 to 70 ppm, respectively. Therefore^27^, A1 NMR is employed to distinguish the two kinds of Al in the clay. As shown in Fig. 6, δ of Al_t_ and Al_o_ of S_0_ was 68.7 and 4.0, both of which were similar to theoretical values. The counterparts of S_2_ turned to be 69.6 ppm and 2.0 ppm, respectively.

According to Fig. 6, the relative ratio of Al_t_ and Al_o_ was calculated for both S_0_ and S_2_ (Table 15). Combining with the data in Table 12, the contents of Al_t_ and Al_o_ in each sample can be known as well. For example, in S_0_, there are 30.02 g Al_2_O_3_ existed in each 100 g of S_0_. Therefore, the amount of Al is 15.89 g and the contents of Al_t_ and Al_o_ are 6.11 g and 9.78 g, respectively. Similarly, there are 3.37 g Al_t_ and 5.73 g Al_o_ existed in 100 g S_2_. The Si/Al_t_ ratio for S_0_ and S_2_ are 3.48 and 10, respectively, which clearly indicates that the fluorosilicate modification by the combination of and nitric acid can improve the Si/Al_t_ ratio in the tetrasheet of sericite. It is speculated that the fluorosilicate modification reduced the layer charge of sercite, leading to weak binding effect of sericite to its interlayer ions, which finally induced the relatively high ion exchange properties of sericite.

**Table 15:** The relative ratio of Al_t_ and Al_o_ in S_0_ and S_2._

	Al_t_		Al_o_		Al_t_ /Al_o_
	δ/ppm	Fraction	δ/ppm	Fraction	
S_0_	68.7	6.25	4.0	10	6.25: 10
S_2_	69.6	5.82	2.0	10	5.82: 10

Based on the above characterization results, the fluorosilicate modification mechanism was proposed as follows: First, when sodium fluorosilicate was used alone to modify the thermal-treated sericite, it had a tiny effect. However, the combination of nitric acid and sodium fluorosilicate had an obvious effect on sericite, which is caused by the strong interactions between the two reagents. The H^+^ in the nitric acid has a strong effect on the structure of sericite and made ion exchanges^40^. It also promotes the effect of fluorosilicate on sericite, probably because fluorosilicate can dissociate much more easily in nitric acid solution and its dissociative products have better acts on sericite. Second, high reaction temperature, low solid-to-liquid ratio, long reaction time, high acid concentration and high fluorosilicate concentration all helped the dissolution of Al^3+^, which are in accordance with the general laws during chemical reaction progress and these conditions can improve the activation energy during the reactions.

Therefore, the mechanism of sodium fluorosilicate to the Al_t_ in sericite was conjectured as follows:

### (1) Fluorosilicate in aqueous solution

First, sodium fluorosilicate dissolved into the solution and made a hydrolysis into Si(OH)_4_ and F^−^.F^−^ combined with the Al_t_ in sericite and dissolved out in the form of ${{\rm{AlF}}}_{6}^{3-}$. The small dissociation degree of fluorosilicate in aqueous solution restricted its degree of dissolving Al from sericite. The related reactions are listed below.

1 $${\rm{N}}{{\rm{a}}}_{2}{\rm{S}}{\rm{i}}{{\rm{F}}}_{6}\to 2{\rm{N}}{{\rm{a}}}^{+}+{\rm{S}}{\rm{i}}{{{\rm{F}}}_{6}}^{2-}$$

2 $${\rm{S}}{\rm{i}}{{{\rm{F}}}_{6}}^{2-}+4{{\rm{H}}}_{2}{\rm{O}}\to {\rm{S}}{\rm{i}}{({\rm{O}}{\rm{H}})}_{4}+6{{\rm{F}}}^{-}+4{{\rm{H}}}^{+}$$

3

### (2) Fluorosilicate in acid solution

The hydrolyzed F^−^ reacted with H^+^ in the solution and generated HF, which reacted with sericite and dissolved large amount of Al from sericite. The relevant reaction is listed below:4

The Si^4+^ and Al^3+^ in sericite have different chemical stabilities and Si^4+^ can hardly be dissolved out. Therefore, the Si/Al ratio increased as the reaction went on, which promoted the reduction of its layer charge.

## Conclusion

In this article, the thermal-treated sericite was modified by both fluorosilicate and the combination of nitric acid and fluorosilicate in order to improve the Si/Al ratio and reduce the layer charge for the preparation of sericite/polymer nanocomposite. Several orthogonal experiments and single factor experiments were done in order to obtain the optimal reaction conditions. After comparison, it is thought that the combination of nitric acid and fluorosilicate has more remarkable effect on sericite than fluorosilicate alone. The optimal experimental conditions are: reaction temperature 90 °C, reaction time 5 h, solid-to-liquid ratio 3 g/200 ml, the volume of nitric acid 10 ml and the concentration of fluorosilicate 0.015 mol/L. The dissolving-out amount of Al^3+^ can reached 65 mg/g and ^27^Al NMR test showed that both Al_t_ and Al_o_ were dissolved out from sericite, while Al_t_ had a lower relative content after modification. After modification, the Si/Al ratio increased from 3.48 to 10 and the layer charge decreased, together with the CEC value increased, which of course, proved the fluorosilicate modification can effectively activate sericite for the preparation of sericite/polymer nanocomposites.
